# Artificial Convolutional Neural Network in Object Detection and Semantic Segmentation for Medical Imaging Analysis

**DOI:** 10.3389/fonc.2021.638182

**Published:** 2021-03-09

**Authors:** Ruixin Yang, Yingyan Yu

**Affiliations:** Department of General Surgery of Ruijin Hospital, Shanghai Institute of Digestive Surgery and Shanghai Key Laboratory for Gastric Neoplasms, Shanghai Jiao Tong University School of Medicine, Shanghai, China

**Keywords:** medical images, convolutional neural network, object detection, semantic segmentation, analysis

## Abstract

In the era of digital medicine, a vast number of medical images are produced every day. There is a great demand for intelligent equipment for adjuvant diagnosis to assist medical doctors with different disciplines. With the development of artificial intelligence, the algorithms of convolutional neural network (CNN) progressed rapidly. CNN and its extension algorithms play important roles on medical imaging classification, object detection, and semantic segmentation. While medical imaging classification has been widely reported, the object detection and semantic segmentation of imaging are rarely described. In this review article, we introduce the progression of object detection and semantic segmentation in medical imaging study. We also discuss how to accurately define the location and boundary of diseases.

## Introduction

**Figure 1:**
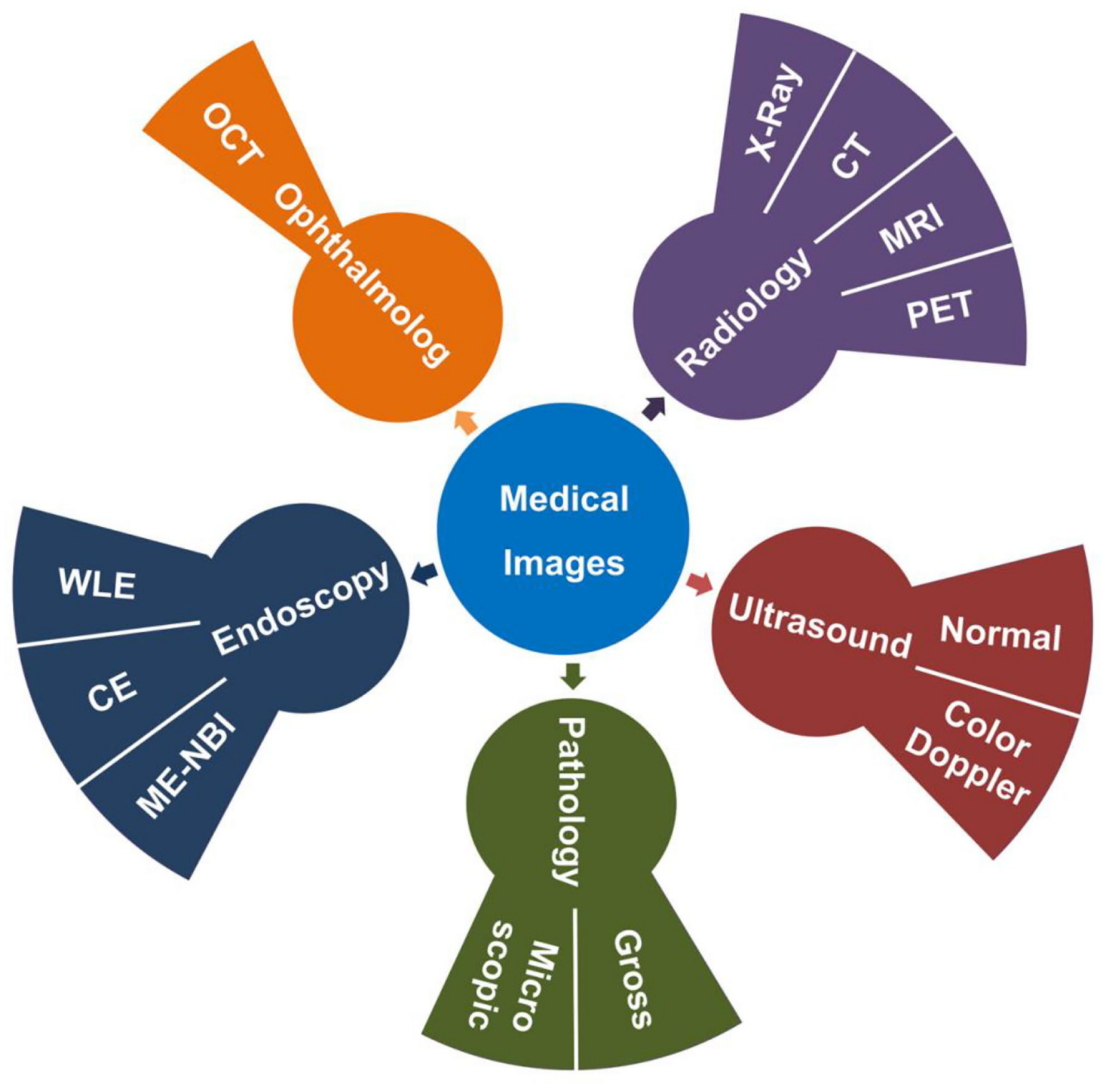
Categories of medical images. The radiological images (Purple) contain X-Ray, CT, MRI, and PET. The ultrasound images (Red) contain normal ultrasound and color Doppler images. The pathological images (Green) include gross images and microscopic images. The endoscopic images (Blue) include WLE, CE, ME-NBI and so on. The images of ophthalmology include OCT (Orange).

In routine medical practice, a large number of medical images are produced in the process of various examinations, such as radiology, ultrasound, endoscopy, ophthalmology, and pathology. Radiation images include X-Ray, computed tomography (CT), magnetic resonance imaging (MRI), and positron emission tomography-computed tomography (PET-CT). The ultrasound images include normal ultrasound images and color Doppler ultrasound images. The endoscopic images contain white light endoscopy (WLE), chromoendoscopy (CE), and magnifying endoscopy-narrow-band imaging (ME-NBI). The images of ophthalmology deal with optical coherence tomography (OCT) images, while the pathological images cover gross images and microscopic images (Figure 1). Clinical doctors have to spend a great deal of time to screen and evaluate these images.

With the development of artificial intelligence (AI), AI industries gradually enter into medical fields, and involve in medical imaging analysis, that help doctors to solve diagnostic problems and improve efficiency (1). AI is a branch of computer science for designing and executing tasks originally carried out by human intelligence (2). Machine learning (ML) is a kind of technologies using computer to perform repetitive and well-defined tasks (3–5). ML includes supervised learning, unsupervised learning, semi-supervised learning, and reinforcement learning. The supervised learning means that the training dataset is labeled by medical experts. The unsupervised learning means that the training dataset is unlabeled. The semi-supervised learning means that a part of training data is labeled and others are unlabeled. The reinforcement learning receives feed-back to obtain the learning information and update the model parameter. Deep learning (DL) is a new direction in ML, which is based on the simulating neural network structure of human brain to build a computational model (5, 6). DL is often used in analysis of high-dimensional data, including image classification, object detection, and semantic segmentation. Convolutional neural network (CNN) is the representative algorithm of DL.

## CNN and Its Extension

The research of CNN could be traced back to 1962, when Hubel and Wiesel analyzed the structure of visual cortex in the cat brain and found that the biological visual information is transferred through multi-layer receptive domain (7). They tried to construct similar algorithms to make the machine recognizes images. The construction and application of CNN developed rapidly after 1980. For instance, Simonyan et al. developed VGG16 (13 convolutional layers and three fully connection layers) and VGG19 (16 convolutional layers and three fully connection layers) (8). Szegedy et al. developed GoogLeNet model with small convolution kernel to reduce the computational complexity by changing convolution modules in 2014 (9). He et al. constructed ResNet model, which accelerates the network convergence speed and improve the image classification effect by increasing the network depth (10). Some new models in combination with different features of the above models were gradually constructed, such as DenseNet and Inception-ResNet-v2, etc. MobileNet is a lightweight CNN model which is introduced by Google in the Conference on Computer Vision and Pattern Recognition in 2017. This model utilized depth-wise separable convolutions to compress model parameters and improve computing speed (11). As a lightweight CNN, MobileNet can be set into mobile equipment to achieve mobile prediction. To optimize the speed and accuracy, Tan et al. introduced EfficientDet, which contains eight model structures, including EfficientDet D 0 to EfficientDet D7 (12).

Image classification is the popular application of CNN algorithms. Recently, scientists tried to integrate traditional CNN algorithms with object detection and semantic segmentation. The purpose of object detection is to make sure whether there are objects from predefined categories. For instance, it can be used to determine the existence and region of tumors on organs or tissues of medical images. If the target is present, it could be indicated on spatial location. The object in images is marked by a frame (like a boundary box) with the confidence on the top of boundary box (13). Object detection can perform many tasks such as lesion location, lesion tracking and image discrimination. The application of object detection in medical images is extremely wide. Semantic segmentation is another algorithm in which computer segments images based on the pixels presented in the images. The semantic refers to the content of the image, and the segmentation means that different objects in the image are segmented based on pixels. In semantic segmentation analysis, each pixel in the image is labeled (14).

## The Common Algorithms for Object Detection

**Figure 2:**
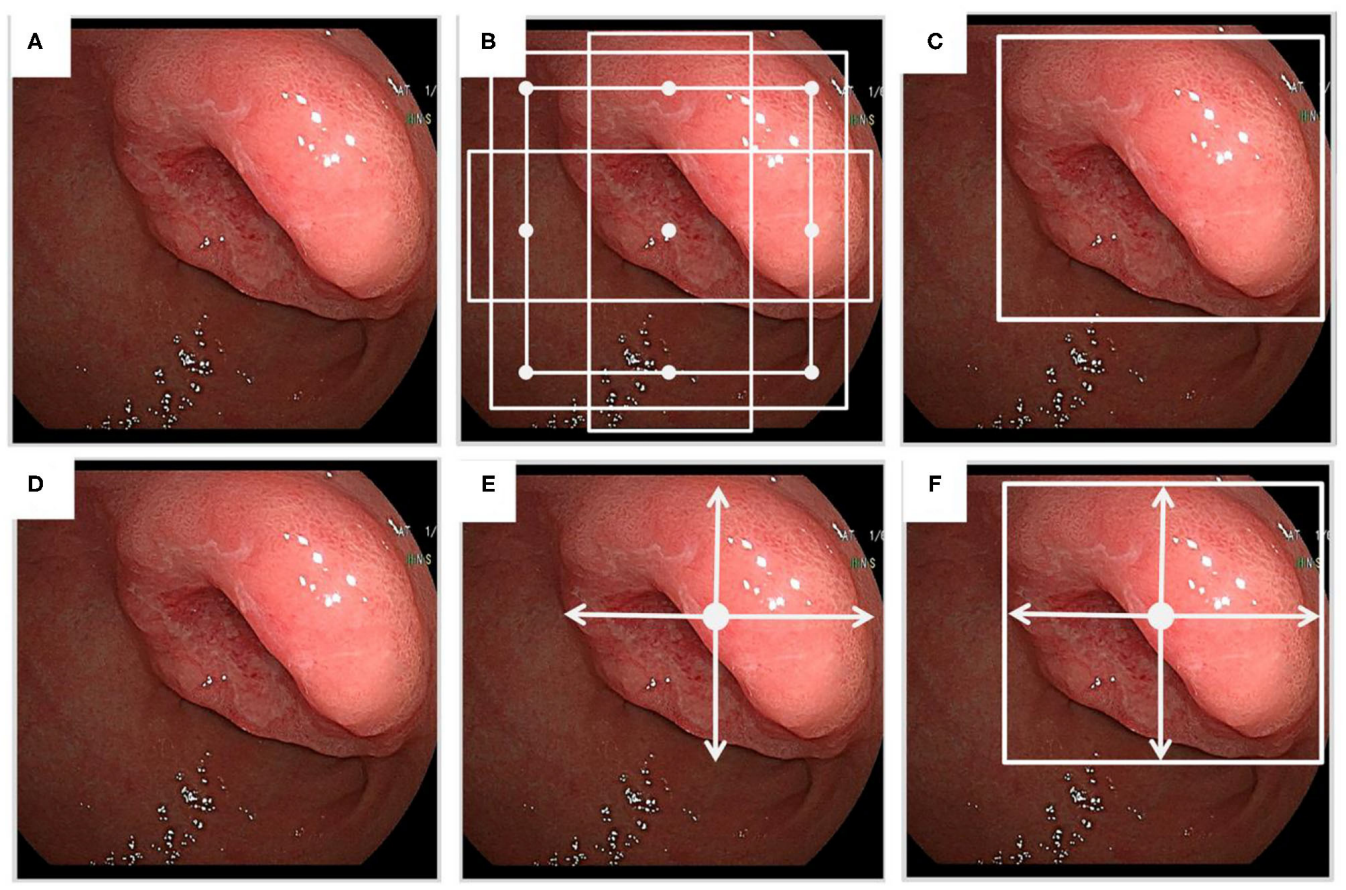
The sketch of object detection. **(A)** A medical image from an endoscopic examination. **(B)** In prior box technology, a group of prior boxes is created during object detection. **(C)** A predicted box is presented. **(D)** The same medical image as above from the endoscopic examination. **(E)** The CenterNet model is used for center point detection. **(F)** Only center point of the object is presented in the predicted box.

Object detection in medical images refers to identify location of lesions and classify different objects. Popular algorithms include R-CNN, Fast R-CNN, Faster R-CNN, PFN, PSPNet, SSD, YOLO, CenterNet, and EfficientNet (15, 16). Object detection has two steps: (1) the target feature extraction, and (2) classifying and positioning the objects. The target feature extraction of image relies on CNN automatically. There are two types of frameworks for object detection: two-stage detection framework, and one-stage detection framework. The former includes a preprocessing step for generating object detection proposal and a step for object detection. The latter has an integrated process containing both steps. The two-stage framework contains two parts. The first part is to extract CNN features of regions from images without category information. The second part is to use category-specific classifier to determine the category labels. The one-stage framework includes SSD, YOLO and CenterNet and EfficientNet series, that are relatively fast but less accurate. Object detection algorithms get the predicted box through the prior box technology, and then adjust parameters of prior boxes to obtain the result of the predicted box. Moreover, the CenterNet algorithm could provide the center point detection by means of predicted box in images (Figure 2).

The R-CNN means the region of CNN, which is based on the framework of AlexNet. The processing of this framework begins from input images. Then the proposed regions are extracted and CNN features are computed to achieve region classification (14). The Fast R-CNN solves some problems of R-CNN, and improves the detecting speed and quality. This framework uses a softmax classifier and class-specific bounding box regression simultaneously. The speed is increased three to ten times in training and testing sets. The Faster R-CNN also utilized CNN to extract features and obtained region of interest (ROI) using region proposal network. The most important improvement of Faster R-CNN is to establish an integrated, simpler, and faster object detection framework relied on CNNs. Lin et al. introduced feature pyramid model into Faster R-CNN to establish feature pyramid network (FPN) to achieve state-of-the-art result without sacrificing speed and internal storage, which is more suitable for small object detection. The backbone network of feature pyramid model utilizes ResNet with three additional parts, including bottom-up pathway, top-down pathway and lateral connection (17). However, the disadvantages of the two-stage framework are the requirement of large resources for computation.

To overcome the above shortcomings, scientists developed the detection strategy of one-stage framework. In one-stage framework, all computation is encapsulated in a single network. YOLO is the abbreviation of You-Only-Look-Once. YOLO solved an object detection problem as a regression problem. The input images are proposed with an inference that enabled the position and category of all objects in the images. YOLO is originated from GoogLeNet containing 24 convolution layers and two fully connection layers. It used a 1 × 1 convolution layer and a 3 × 3 convolution layer to replace the Inception structure (18, 19). Single Shot Multibox Detector (SSD) (15) is faster than YOLO and competitive with region-based detectors such as Faster R-CNN with a higher accuracy. SSD inherits the method of transforming detection to regression, and completes region proposals and classification in one stage. It improves the running speed and detection accuracy compared with other frame works. Most of the one-stage framework can adjust parameters of prior boxes to show all potential object locations and classify objects in detection results. This detecting method takes too much time and reduces detection efficiency. The CenterNet model achieves improvement in speed and accuracy. The key-point estimation is utilized in CenterNet to find the central point and go back to other object properties that do not need detection of all potential objects with high accuracy. Whereas, the EfficientDet designed by Tan et al. used EfficientNet as backbone, and constructed bi-directional feature pyramid network (BiFPN) to obtain continuous fusion of up-sampling and sub-sampling (12).

## The Common Algorithms for Semantic Segmentation

Before deep learning was applied to computer vision, researchers always use TextonForest or Random Forest to construct the classifier for semantic segmentation. With the development of deep learning, especially the emergence of CNN, computer algorithms with deep learning not only classify images accurately, but also perform better work on segmentation. It is known that images composed of many pixels. In the task of image semantic segmentation, computer algorithms segment images based on the semantic and pixels presented in images. The input is a three-channel RGB image of H × W × 3 and the output is a corresponding H × W matrix whose element indicated the semantic label in the corresponding pixel. The analytic results of semantic segmentation not only identify the objects, but also mark the boundaries of each object. The current popular algorithms for semantic segmentation include FCN, SegNet, PSPNet, DeepLab and UNet (20–22). Fully Convolutional Network (FCN) uses the fully convolutional network for the end-to-end training of segmentation. FCN modifies the structure of VGG16 and other networks to generate segmented images of the same size with an input of non-fixed size. The most important change of FCN is that all fully connected layers are replaced by convolutional layers. The structure of FCN includes convolution, upsampling and skip connection. FCN can accept input images of any size and avoid repetition of storing and calculating the convolution. In 2015, Badrinarayanan et al. proposed SegNet, a new framework of semantic segmentation (23). This framework is based on FCN and constructs an encoder-decoder structure based on VGG16. It is the first time to utilize symmetric network structure and had good performance on semantic segmentation. Based on FCN, PSPNet utilizes pre-trained ResNet101 as the feature extraction layer and introduces the pyramid pooling module to identify the prior information of the context in the image. It has fantastic understanding and higher identification of complex scenes (24). Ronneberger et al. proposed U-Net algorithm for semantic segmentation in 2015. In UNet analysis, two steps of feature extraction, sub-sample and up-sample are needed. Since the network structure is like the letter “U,” it is called UNet (22). The UNet model is simple with few parameters. It is suitable for the classification of medical images with small dataset. In medical imaging analysis, it is easy to overfit if more parameters are involved in the model. Therefore, the UNet model performs well in most medical imaging analysis. To improve the efficiency, Zhou et al. introduced UNet++, a nested UNet architecture for medical image segmentation. UNet++ borrowed the dense connection of DenseNet and improved the skip connection structure of UNet (25).The VNet is constructed to satisfy the need of analyzing 3D images in CT and MRI images, that have similar structure of UNet (26). In DeepLab framework (DeepLab v1), atrous convolution was used in combination of CNN for semantic segmentation. To optimize performance, DeepLab v2 added a new model, atrous spatial pyramid pooling (ASPP), which utilized atrous convolution to get multi-scale information and reduces computation instead of fully connection layer. And DeepLab v3 improved the ASPP model with one 1 × 1 convolution and three 3 × 3 convolution. This framework is a genetic framework which can be applied to any network such as VGG, and ResNet. For DeepLab v3, a simple and efficient decoder model was designed to improve segmentation results (21, 27).

## The Performance Comparison of Available Algorithms

There are a large number of open-source packages for running CNN programs. The convolutional architecture for fast feature embedding (Caffe) was born in Berkeley, California and now hosted by Berkeley Vision and Learning Center (BVLC). Caffe is an early framework with high-performance and seamless switching between CPU and GPU models. It supports cross-platform of Windows, Linux and Mac (28). With the emergence of Tensorflow and Pytorch, Caffe is not the first choice any more. Tensorflow is the open resource of Google at November, 2015, and then updated to Google TensorFlow 1.0 in 2017 (29). Keras is a re-encapsulation of Tensorflow to support a fast practice allowing researchers to quickly turn ideas into results (30). Pytorch is the python version of torch, a neural network framework that is specifically targeted at GPU-accelerated deep artificial neural network programming. Comparing with Caffe and Tensorflow, Pytorch has become the most popular framework in 2019. As an open-source framework by Facebook, Pytorch is compact, easy to use and supports dynamic graphs (31).

**Figure 3:**
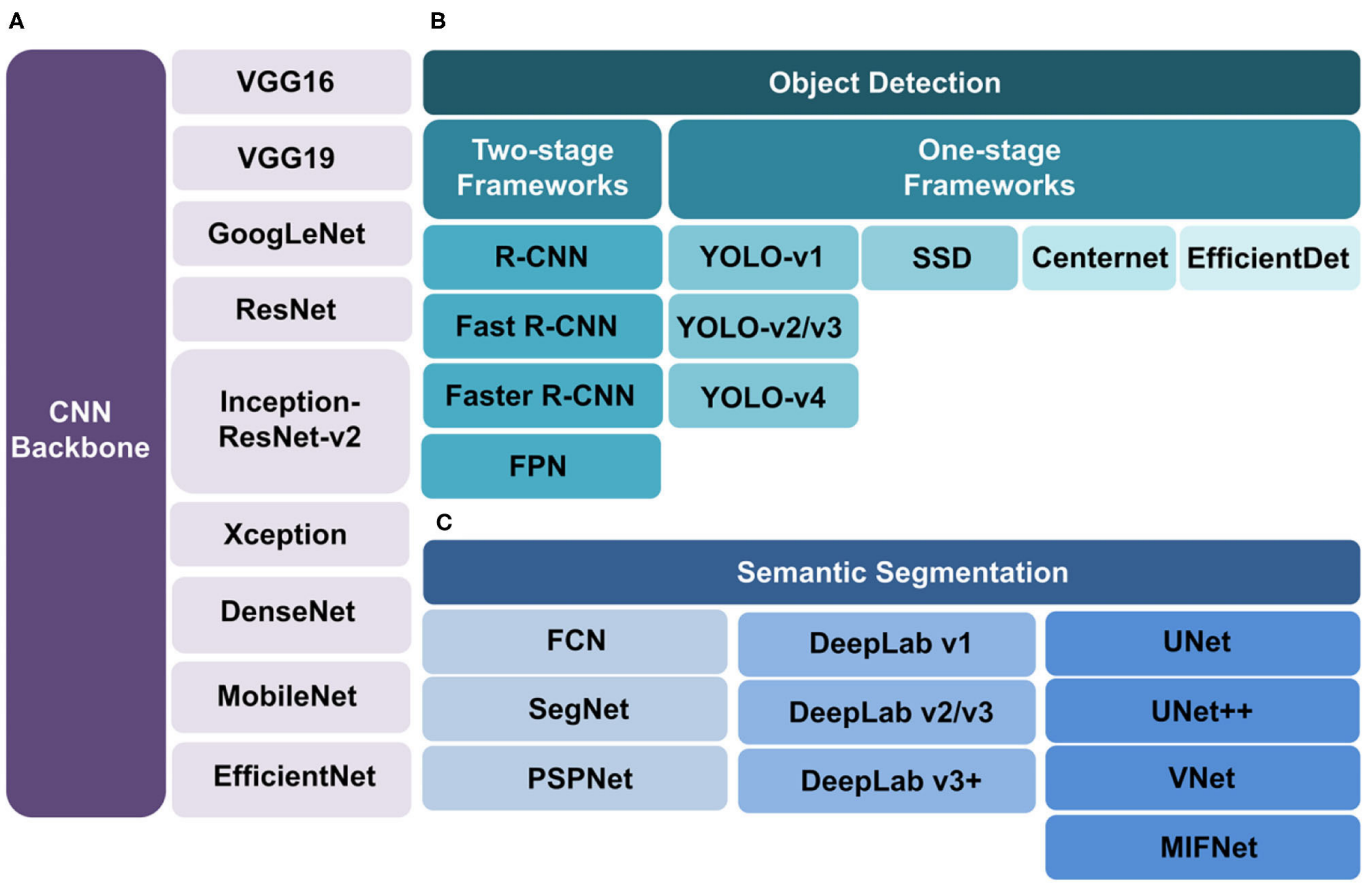
Algorithms of CNN backbone, object detection and semantic segmentation. **(A)** CNN backbones. To achieve feature extraction in medical images, many CNN models can be selected such as VGG16, VGG19, GoogLeNet, ResNet, Inception-ResNet-v2, Xception, DenseNet, MobileNet, and EfficientDet. **(B)** Object detection algorithms include two types. The two-stage framework (left branch) includes R-CNN, Fast R-CNN, Faster R-CNN and FPN with high accuracy. The one-stage framework (right branch) includes SSD, YOLO, CenterNet and EfficientDet with fast speed but low accuracy. **(C)** Semantic segmentation algorithms are divided into FCN, DeepLab, and UNet series. FCN (left branch) is the first algorithm which uses fully convolution network without fully connection layers. SegNet and PSPNet are based on it. DeepLab (middle branch) is a novel algorithm under development. UNet (right branch) is the most popular algorithm. The UNet++, VNet and MIFNet are the derivatives of UNet.

The performance of object detection and semantic segmentation algorithms is highly dependent on the data. To avoid overfitting, some image augmentation methods could be used to ensure input sufficient data size including flipping, cropping, rotation, translation, noise injection, random erasing, mixing images and so on (32).The advantages and disadvantages of the above introduced algorithms of object detection and semantic segmentation (Figure 3) are listed in Table 1.

**Table 1:** Advantages and limitations of object detection and semantic segmentation models.

**Purpose**		**Models**	**Advantages**	**Limitations**
Object detection	Two-stage framework	R-CNN	CNN extracts features automatically.	Extract features in every object box using CNN; Slow speed and low accuracy.
		Fast R-CNN	Single feature extraction by CNN. Fast and high accuracy.	Extract ROI by selective searching. Slow.
		Faster R-CNN	Adding RPN to extract ROI. The object detection rate is high.	Detecting whole image by RPN with slow speed.
		FPN	Adding feature pyramid model, and good for small object detection.	Slow speed comparing with one-stage framework.
	One-stage framework	YOLO	Based on GoogLeNet, fast in speed.	Bad performance for small object detection. More parameters and higher occupation of GPU than SSD.
		SSD	Balance advantages of YOLO and Faster R-CNN with high detection speed and high object detection rate.	Bad performance in small object detection comparing with Faster R-CNN.
		CenterNet	The balance of speed and accuracy. Using the key-point estimation to find the central point.	Difficult to deal with the coincidence of two object centers.
		EfficientDet	Introducing BiFPN to obtain continuous fusion of up-sampling and sub-sampling.	Parameter setting relys on experience.
Semantic segmentation		FCN	Becoming full convolutional layer (without connected layer).	Low accuracy of feature maps with high GPU occupation.
		SegNet	The first symmetric network.	Slow speed.
		UNet	The structure is simple like the letter U with less parameter. Suitable for object detection in small number of medical images.	Difficult to obtain uniform standard of sub-sampling and up-sampling.
		DeepLab	Using atrous convolutional layer.	The atrous convolution layer occupied high GPU.
		PSPNet	Using the Pyramid pooling module to identify the prior information; Fantastic understanding and high identification of complex scenes.	Base backbone of ResNet101 made processing speed slow.

The performance of deep learning algorithms could be evaluated by several parameters. Researchers optimize their models by the indexes of accuracy, specificity, sensitivity, recall, receiver operating characteristic curve (ROC), and area under curve (AUC). As the specific indexes to evaluate the training results, in the field of object detection, mean average precision (mAP) is introduced. The AP value is presented by a curve according to all precision values and recall values. The horizontal coordinate represents the recall value, and the vertical coordinate represents the precision value. The region under the curve is the AP value of one class. The mAP value means the AP average of all classes. In semantic segmentation algorithm, intersection over union (IoU) is used to evaluate the testing results. IoU refers to the ratio of predicted region and marked region. The higher the IoU value, the better the model.

## The Application of Object Detection in Medical Image Analysis

Different types of algorithms can be applied in different medical image analyzing. Endoscopy is an essential tool for the diagnosis of digestive diseases. Endoscopy makes lesions of the digestive tract visible and biopsies can be taken for histology. It is often used for early diagnosis or follow-up of cancers postoperatively. However, inexperienced doctors may overlook some atypical lesions because most of those lesions arise from atrophic mucosa that results in false-negative results. The object detection algorithm could detect lesions automatically and assist diagnosis during the process of endoscopic examination. Hirasawa et al. used SSD to diagnose gastric cancer in chromoendoscopic images. The training dataset consisted of 13,584 images and the test dataset included 2,296 images from 77 gastric lesions in 69 patients. The SSD performed well to extract suspicious lesions and evaluate early gastric cancer. The result showed that the time spent for analyzing 2,296 images is 47 s, and the total sensitivity was 92.2%. It meant that SSD model could analyze a large number of endoscopic images in a short period of time, and greatly reduced the load of endoscopic doctors (33). Wu et al. proposed an object detection model-ENDOANGEL for real-time gastrointestinal endoscopic examination. The ENDOANGEL can efficiently extract suspicious lesions and evaluate the severity of lesions. ENDOANGEL has been utilized in many hospitals in China for assisting clinical diagnosis (34). Gao et al. analyzed peri-gastric metastatic lymph nodes of CT images using Faster R-CNN. The analysis was divided into two stages, the initial learning stage for training and the precise learning stage for fine-tuning and testing. The result showed that, in the initial learning stage, the recall rates of nodule classes for training set and validation set, the mAP was 0.5019 and AUC was 0.8995. In the precise learning stage, the mAP and AUC were 0.7801 and 0.9541, which was obviously improved, compared to initial learning stage. Thus, the Faster R-CNN model had high judgment effectiveness and recognition accuracy for CT diagnosis of peri-gastric metastatic lymph nodes (16).

## The Application of Semantic Segmentation in Medical Image Analysis

For accurate delineation of lesions border, sematic segmentation based on CNN backbones has a potential application. This series of algorithms of DeepLab provide a great choice for accurate delineation of tumor margin. In the examination of cancers, accurate delineation of tumor margin is critical for the choice of treatment and surgical resection, especially when the resection is performed under the endoscope. Luo et al. developed a Gastrointestinal Artificial Intelligence Diagnostic System (GRAIDS) based on DeepLab v3+ for diagnosis of upper gastrointestinal cancers in endoscopic images. After input the endoscopic images of the upper gastrointestinal tract cancers, the model provides two outputs, a standard two-class task for lesion classification and a semantic segmentation task capturing the tumor regions. The accuracy of using this system was 95.5% in the internal validation dataset, 92.7% for the prospective validation dataset, and 91.5 to 97.7% for the external validation dataset. The diagnostic sensitivity of GRAIDS was similar to endoscopic experts and superior to non-expert endoscopic doctors (35).

UNet and its extension models are a series of algorithms to achieve semantic segmentation in medical field. An et al. reported that UNet++ model can delineate the resection margins of early gastric cancer under the indigo carmine chromoendoscopy or white light endoscopy (36). Besides accurate delineation of tumor margin, for beast MRI images, Piantadosi et al. aimed to construct and modify a DCNN model based on UNet to achieve 3D MRI image segmentation of breast parenchyma from other tissues. There were two datasets, the first dataset was a private dataset and the second one was a public breast MR image dataset. After training and testing, the result showed that the modified model performed better and the median dice similarity coefficient (DSC) for both the datasets was 96.60 and 95.78% (37). At present, the contradiction between a large number of pathological images and a shortage of pathologists was a problem worldwide. There is a great opportunity in the field of pathology for deep learning algorithm. Cai et al. constructed a multi-input model called MIFNet to segment lesions in pathological images, and increase the dice coefficient to 81.87%. That was a great progress because the dice coefficient in some existing segmentation models was relatively low, i.e., 67.73% in UNet, and 63.89% in SegNet. They believed that semantic segmentation algorithm was suitable for analyzing pathological images (38). In addition, the ENDOANGEL model not only realized automatic object detection during endoscopic examination, but also realized semantic segmentation. An et al. reported that UNet++ model can delineate the resection margins of early gastric cancer under the indigo carmine chromoendoscopy or white light endoscopy (36). Wickstrømand et al. utilized semantic segmentation models of FCN, UNet and SegNet to analyze endoscopic images of colorectal polyps. The result showed that the FCN performed better than other two models (39).

## Conclusion

**Figure 4:**
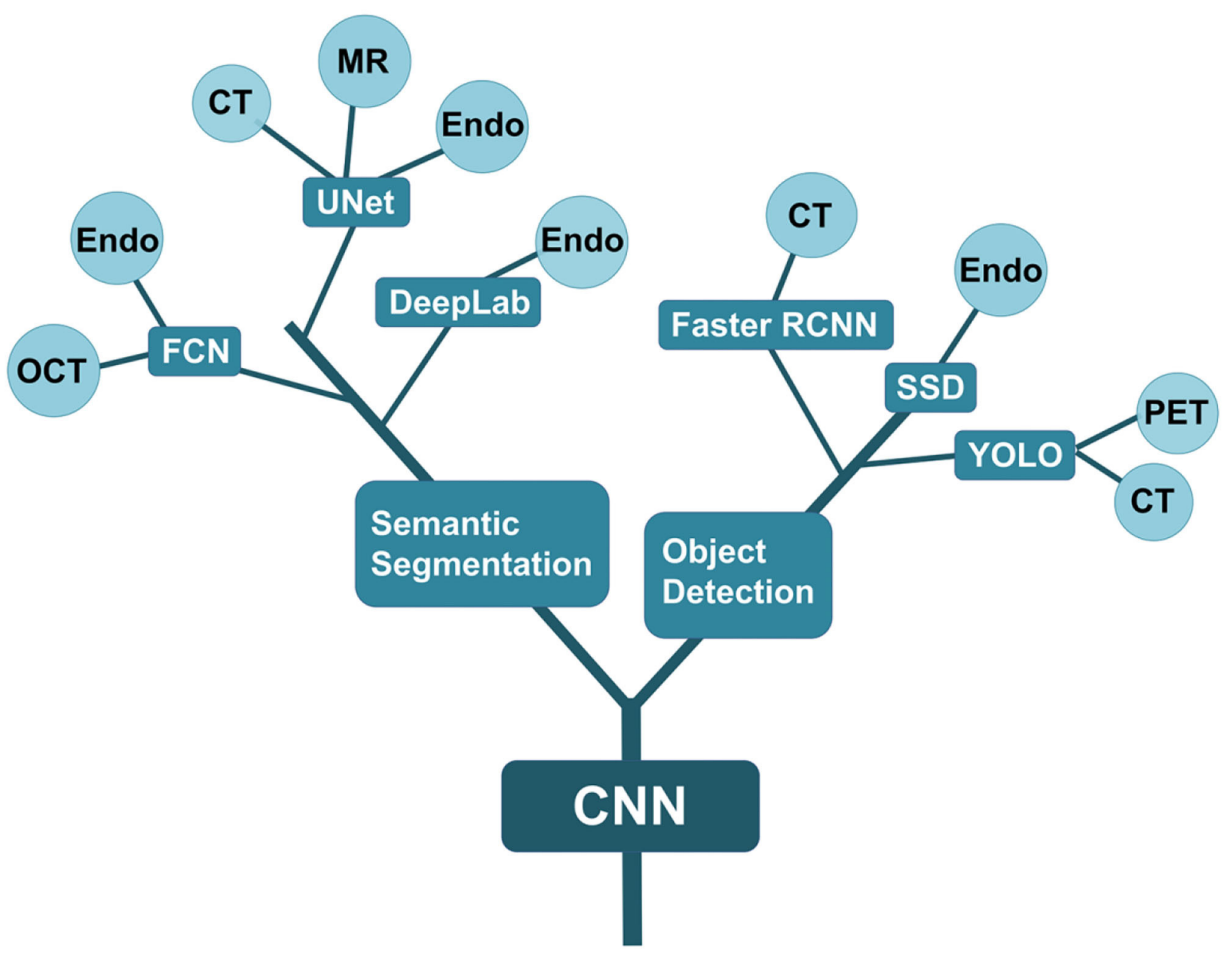
Evolutional tree of AI algorithms for medical image analysis. The CNN model is the backbone algorithm. And then, object detection and semantic segmentation are developed. The two algorithms are further divided into different branches including FCN, UNet and DeepLab, faster RCNN, SSD, and YOLO. The ends of each branch correspond to the application in various medical images. Endo refers to endoscopic images. OCT means the images of optical coherence tomography.

Both object detection and semantic segmentation algorithms are based on CNN. They are widely applied in various fields of medical imaging study, particularly in the digestive system, respiratory system, endocrine system, cardiovascular system, brain, eye, and breast. These algorithms can be used to analyze multiple images including radiation images (CT, MRI, and PET), pathological images, ultrasound images, and endoscopic images. The development of various algorithms and their application are presented in Figure 4.

However, there are some limitation of object detection and semantic segmentation in the application of analyzing medical images. In the model training stage, a large number of medical images are needed. In addition, both object detection and semantic segmentation belongs to supervised algorithms, which require experienced doctors to label images. Therefore, future study should focus on how to use limited medical images to get good training results.

## Author Contributions

YY and RY designed the study. RY wrote the manuscript. YY reviewed and revised the manuscript. All authors contributed to the article and approved the submitted version.

## Conflict of Interest

The authors declare that the research was conducted in the absence of any commercial or financial relationships that could be construed as a potential conflict of interest.
